# Secreted Phospholipases A_2_ from Animal Venoms in Pain and Analgesia

**DOI:** 10.3390/toxins9120406

**Published:** 2017-12-19

**Authors:** Vanessa O. Zambelli, Gisele Picolo, Carlos A. H. Fernandes, Marcos R. M. Fontes, Yara Cury

**Affiliations:** 1 Laboratory of Pain and Signaling, Butantan Institute, Av. Vital Brasil, 1500, 05503-900 São Paulo, SP, Brazil; vanessa.zambelli@butantan.gov.br (V.O.Z.); gisele.picolo@butantan.gov.br (G.P.); 2 Department of Physics and Biophysics, São Paulo State University (UNESP), Rua Professor Doutor Antonio Celso Wagner Zanin, s/n, 18618-689 Botucatu, SP, Brazil; fernandes@ibb.unesp.br

**Keywords:** secretory phospholipases A_2_, catalytic activity, animal venoms, pain, analgesia

## Abstract

Animal venoms comprise a complex mixture of components that affect several biological systems. Based on the high selectivity for their molecular targets, these components are also a rich source of potential therapeutic agents. Among the main components of animal venoms are the secreted phospholipases A_2_ (sPLA_2_s). These PLA_2_ belong to distinct PLA_2_s groups. For example, snake venom sPLA_2_s from Elapidae and Viperidae families, the most important families when considering envenomation, belong, respectively, to the IA and IIA/IIB groups, whereas bee venom PLA_2_ belongs to group III of sPLA_2_s. It is well known that PLA_2_, due to its hydrolytic activity on phospholipids, takes part in many pathophysiological processes, including inflammation and pain. Therefore, secreted PLA_2_s obtained from animal venoms have been widely used as tools to (a) modulate inflammation and pain, uncovering molecular targets that are implicated in the control of inflammatory (including painful) and neurodegenerative diseases; (b) shed light on the pathophysiology of inflammation and pain observed in human envenomation by poisonous animals; and, (c) characterize molecular mechanisms involved in inflammatory diseases. The present review summarizes the knowledge on the nociceptive and antinociceptive actions of sPLA_2_s from animal venoms, particularly snake venoms.

## 1. Introduction

Animal venoms comprise a complex mixture of components that affect several biological systems. Based on the high selectivity for their molecular targets, these components are also considered a rich source of potential therapeutic agents [1,2]. One of the main components of animal venoms are the secreted phospholipases A_2_ (PLA_2_s-EC 3.1.1.4) that hydrolyze the acyl bond at the sn-2 acyl position of membrane phospholipids, releasing lysophospholipids and free fatty acids, such as arachidonic and oleic acids [3,4].

The products of phospholipid hydrolysis constitute precursors of signaling molecules that take part in many biological processes. Arachidonic acid molecules, for example, can be converted to eicosanoids, including prostaglandins and leukotrienes, which participate in a wide range of physiological and pathological processes, such as sleep regulation, immune responses, inflammation, and pain [5]. Furthermore, the lysophospholipids can serve as precursors for lipid mediators, such as lysophosphatidic acid (LPA) or platelet activating factor (PAF). LPA acts as a potent signaling molecule with a wide range of effects, including cell proliferation, survival, and migration, in many different target tissues [6,7]. Despite the important role of the LPA signaling in organism development and physiological functions, this pathway contributes to several human diseases, including cardiovascular diseases, cancer, neuropathic pain, neuropsychiatric disorders, reproductive disorders, and fibrosis [7]. PAF signaling cascade evolved as a component of innate host defense, but it is also involved in inflammation, anaphylaxis, and thrombotic diseases [8].

Currently, the superfamily of PLA_2_s comprises a number of proteins that are classified in fifteen groups and can also be divided into five distinct types: secreted PLA_2_s (sPLA_2_s), cytosolic PLA_2_s (cPLA_2_s), Ca^2+^-independent PLA_2_s (iPLA_2_s), PAF acetylhydrolases (PAF-AH), and lysosomal PLA_2_s [9]. The sPLA_2_ group is composed by small proteins (14–18 kDa), usually containing five to eight disulfide bonds. This group has sixteen subgroups, and includes the phospholipases A_2_ from snake and bee venoms, which display several pharmacological effects, such as pre- [10] or post-synaptic neurotoxicity [11], myotoxicity [12], cardiotoxicity [13], bactericidal [14], platelet aggregation inhibition [15], edema [16], anti-coagulation [17], convulsion [18], and hypotension [19]. Furthermore, numerous studies have been highlighting the role of these toxins in inflammation and pain. The present review summarizes the knowledge on the nociceptive and antinociceptive actions of sPLA_2_s from animal venoms, particularly from snake venoms.

## 2. Animal Venom sPLA_2_s

Venom phospholipases A_2_ (svPLA_2_s) from Elapidae and Viperidae (pit vipers) families—the most important snake families when considering envenomation and their effects—belong, respectively, to IA and IIA/IIB groups [9]. This classification is based on sequence similarity, position of disulfide bonds and loops insertions. The snake venom sPLA_2_s classified in groups IA and IIA have seven disulfide bonds—six of them are conserved in both Elapidae and Viperidae families, whereas the disulfide bonds Cys11/Cys77 and Cys51/Cys133 are only found in, respectively, elapids and viperids. Moreover, the group IIB svPLA_2_s have only six disulfide bonds, lacking the Cys61/Cys91 bond [9,20,21]. Interestingly, PLA_2_ from human synovial fluid, which is present in high concentration in inflammatory conditions, such as arthritis, are also included in the IIA group due to it structural similarity to svPLA_2_s [9].

**Figure 1:**
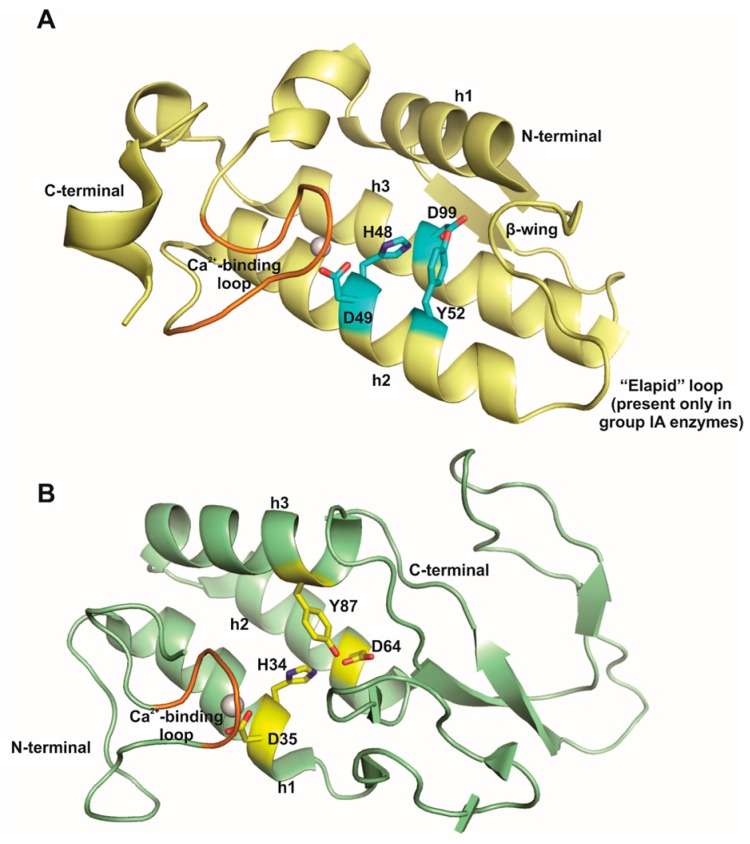
Cartoon representation of canonical tertiary structure of phospholipases A_2_ from (**A**) snake venoms of groups I and II; and, (**B**) bee venom of group III. The conserved structural motifs are highlighted: α-helices 1, 2, and 3 (h1, h2 and h3, respectively); the β-wing, the flexible C-terminal region, the Ca^2+^-binding loop (in orange) and the “elapid” loop, an insertion of two or three amino acids in region 52–65 present only in class IA enzymes. The conserved catalytic network formed by a histidine (H), two aspartic acids (D) and a tyrosine (Y) residues are also highlighted in cyan (in snake venom PLA_2_) and in yellow (in bee venom PLA_2_) sticks. The figures were generated using the crystal structures of group IA PLA_2_ from *Naja naja* venom (PDB ID 1PSH) and of group III PLA_2_ from *Apis mellifera* venom (PDB ID 1POC). Modified from Fernandes et al., 2014: Biochimica et Biophysica Acta (BBA)-Preoteins and Proteomics, volume 1844, pages 2265-2276, Elsevier, copyright 2014 [23].

The tertiary structure of snake venom sPLA_2_s is highly conserved [20]. The canonical snake venom sPLA_2_ (Figure 1a) includes a motif conserved in all class I/II enzymes, defined by two long antiparallel α-helices (helices 2 and 3, residues 37–53 and 90–109; respectively), linked by disulphide bonds in conjunction to a highly conserved Ca^2+^-binding loop (^26^CGYCGXGGXG^35^) [20,22]. The Y residue at position 28 has an essential role in Ca^2+^ binding, due to electrostatic interaction between the Oγ of Y28 and G35 amino group that stabilizes the Ca^2+^-binding loop [22].

Although these two α-helices do not display a clear amphipathic character, the hydrophilic amino acid side chains are generally exposed to the solvent and the hydrophobic residues point into the protein core (which constitutes the hydrophobic channel of the protein). The only non-hydrophobic residues located in the protein core, which form the catalytic network, are H48, D49, Y52, and D99. The other conserved structural features are the N-terminal helix, the β-wing region (formed by a small anti-parallel β-sheet), the Ca^2+^-binding loop, the “elapid loop” (an insertion of two or three amino acids in region 52–65 present only in class IA enzymes), the short helix and the C-terminal loop, a very flexible region that can adopt different relative orientations [20].

Bee venom phospholipase A_2_, classified as group III of secreted phospholipases A_2_, has eight disulfide bonds [9]. There is only one crystal structure of bee venom PLA_2_ (PDB ID 1POC), in contrast to the vast number of PLA_2_ crystal structures from class I/II PLA_2_ in PDB Data Bank (about forty structures). Structurally, bee venom PLA_2_ conserves the catalytic network and presents functional substructures that are found in class I/II enzymes, however, they are arranged with a different overall architecture [24]. Bee venom PLA_2_ presents shorter α-helices, a Ca^2+^-binding loop closer to the active site in the N-terminal region (residues 7–14) and a higher content of β-sheets when compared to group II crystal structures (Figure 1b). The Ca^2+^-binding loop conserves the Ca^2+^ binding motif (^8^XCGXG^12^) and a W residue in position 8 replaces the above-mentioned conserved Y in class I and II enzymes [24].

Currently, there are two proposed catalytic mechanism for secreted PLA_2_s—the single-water and assisting-water mechanisms. The single-water mechanism was proposed upon crystallographic structures of a snake venom PLA_2_ from class I and a class III bee venom PLA_2_ complexed to transition-state analogues [25]. In this mechanism, the His48 Nδ1 atom is stabilized by the carboxyl oxygen atom of Asp99, which is hydrogen that is bound to the hydroxyl oxygen atom of Tyr52. After phospholipid binding, the His48 Nδ1 atom abstracts a proton from a structurally conserved water molecule, initiating the nucleophilic attack of *sn*-2 position of the substrate, forming a tetrahedral oxyanion intermediate [25]. This tetrahedral intermediate is stabilized by the Ca^2+^ cofactor, which, in its turn, is kept in position due to interactions with the carboxyl oxygen atom(s) of Asp49, carbonyl main chain oxygen atoms of the Ca^2+^ binding loop, and two structurally conserved solvent water molecules [25]. A disulfide bond ensures the correct relative orientation of the calcium-binding loop in relation to the amino acids that form the catalytic region. Upon collapse of the tetrahedral intermediate and release of hydrolysis products, three water molecules move into the active site [25]. In the assisting-water mechanism, a Ca^2+^-coordinated water, which is hydrogen-bonded to His48 through a second water molecule, is the responsible for the nucleophilic attack [26,27]. Remarkably, the assisting-water mechanism requires two tetrahedral intermediates and the first PLA_2_ crystal structures that was used to propose the single water mechanism are entirely consistent with the second tetrahedral intermediate of the assisted-water mechanism [26,27,28]. A helpful schemecomparing the two proposals for the catalytic mechanisms was published before by Bahnson, 2005 (Supplementary Figure S1) [26,27,28]. Despite possible differences between these mechanisms, several studies demonstrated the Ca^2+^ ion is an obligatory cofactor for secreted phospholipases A_2_ catalysis. Studies of its substitution by other divalent ions showed that Cd^2+^, Sr^2+^, Ba^2+^, Mg^2+^ do not support catalytic hydrolysis; however, Cd^2+^ is still able to keep the substrate bound to the enzyme [29]. On the other hand, Ni^2+^ and Co^2+^ ions support a significant catalytic hydrolysis of phospholipid with specific head groups, indicating a plasticity of the active site environment [27].

In addition to the catalytic site, the interaction of secreted phospholipases A_2_ with phospholipids is critical for their activity [3,30]. These PLA_2_s must establish specific interactions along their interface-binding surfaces (i-face) in order to reach their activated forms on phospholipids interface [30]. Both electrostatic and hydrophobic interactions contribute to the interfacial binding of sPLA_2_s to phospholipid membranes [3,28]. Snake venom PLA_2_s from group I can hydrolyze zwitterionic phospholipids due to the presence of the aromatic residues Y3, W61, Y63, and F64 on their i-face [31]. These residues penetrate into the lipid membrane phase to allow the enzyme to access the substrate from the membrane lipids [31]. Regarding anionic phospholipids, the K hydrophilic residues in 6 and 10 positions establish electrostatic interactions with this anionic interface [31]. Regarding snake venom PLA_2_s from group II, the aromatic Y residues, cationic hydrophilic K and R residues, and hydrophobic L and F residues are responsible for electrostatic and hydrophobic interactions to the interfacial binding of these proteins on membranes [32,33]. On the other hand, studies on the i-face of group III PLA_2_ from bee venom have shown that its interaction with membrane phospholipids occurs predominantly through a non-electrostatic mechanism [34]. The mutation of five R/K basic residues on bee venom group III-PLA_2_ to all of the neutral glutamine residues resulted in no significant decrease in binding to anionic vesicles. However, if these basic residues are mutated to charge-reversed glutamate residues, there is a high decrease of mutant binding to those vesicles [34]. These data indicate that, although electrostatic interactions are not predominant between the bee venom group III-PLA_2_ and anionic phospholipids, the repulsion interaction will definitely impair the binding [34].

## 3. Pain and Analgesia: General Concepts

Envenomation is frequently associated with pain, and venom secretory PLA_2_s have been a useful tool for the understanding of this phenomenon. In contrast, some venom neurotoxic sPLA_2_s are able to inhibit pain by mechanisms involving the endogenous systems of pain control. Before discussing how sPLA_2_s from animal venoms contribute to pain and its control, it is important to review how the pain pathways work.

The International Association for the Study of Pain (IASP) defines pain as “an unpleasant sensory and emotional experience associated with actual or potential tissue damage, or described in terms of such damage. Pain is always subjective. Each individual learns the application of the word through experiences that are related to injury in early life”. Because pain perception is always subjective, the term nociception is used for the neural process of encoding and processing noxious stimuli [35]. Noxious stimuli, such as high temperatures, injury-related chemicals, and extreme mechanical pressures, are detected by specialized peripheral sensory neurons, called nociceptors. There are also the “silent” nociceptors that are unresponsive to noxious intensities of mechanical stimulation, except at extreme ranges of intensity [36].

**Figure 2:**
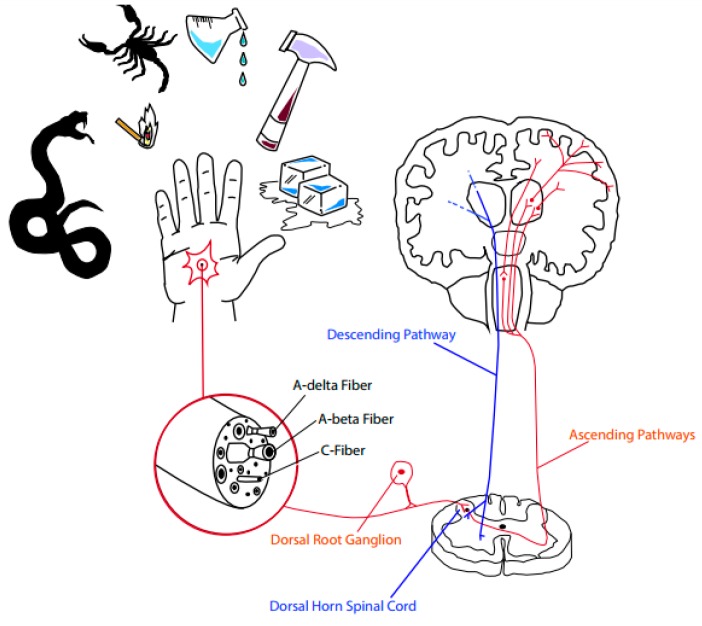
A schematic overview of pain pathways. Noxious stimuli, such as high temperatures, injury-related chemicals, extreme mechanical pressures and venoms, are detected by nociceptors. The nociceptors are pseudounipolar neurons whose cell bodies (soma) are located in the dorsal root ganglia. They bifurcate, sending a peripheral axon to the skin, and other organs, and an axon to the central nerve system (CNS). C-fibers are unmyelinated small diameter axons with projections to superficial laminae I and II of the dorsal horn of the spinal cord. Aδ-fiber nociceptors are thinly myelinated axons with projections to superficial lamina I as well as to the deeper dorsal horn (lamina V). From the spinal cord the information proceeds to the brainstem and reaches the cerebral cortex, where the perception of pain occurs (ascending pathways-red). The descending pain modulatory circuits decrease the nociceptive input in the central nervous system by releasing neurotransmitters that can exert an inhibitory action (ascending pathways-blue). Illustration: Larissa Foronda.

The nociceptors are pseudounipolar neurons whose cell bodies (soma) are located in the peripheral ganglia (dorsal root ganglia for the body and the trigeminal ganglia for the face). They bifurcate, sending a peripheral axon to the skin, and other organs, and an axon to the central nerve system (CNS). C-fibers are unmyelinated small diameter axons with projections to superficial laminae I and II of the dorsal horn of the spinal cord. Aδ-fiber nociceptors are thinly myelinated axons with projections to superficial lamina I, as well as to the deeper dorsal horn (lamina V). From the spinal cord the information proceeds to the brainstem and reaches the cerebral cortex, where the perception of pain occurs [37] (Figure 2).

The noxious stimuli are converted to electrical activity by transient receptor potential-generating channels (TRP channels) and purinergic channels, and this electrical activity is amplified by sodium channels to elicit action potentials. Although physiological pain has as a protective function, it can become pathological in certain conditions, such as neuropathy and inflammation. In these conditions, individuals and animals experience an increased sensitivity to painful stimuli (hyperalgesia). Individuals with neuropathic pain often show allodynia (pain induced by a non-noxious stimulus, such as light touch) and spontaneous pain. In this review, we will focus on inflammatory pain as it is an usual symptom of envenomation [38].

Upon tissue damage, nociceptors or non-neural cells that reside within or infiltrate into the injured area (including mast cells, neutrophils, endothelial cells, basophils, platelets, fibroblasts, macrophages, and keratinocytes) release signaling molecules, such as neuropeptides (substance P, CGRP), bradykinin, cytokines, chemokines, neurotrophins, nitric oxide, proteases, protons, and reactive aldehydes, as well as extracellular PLA_2_-derived lipid mediators [39,40]. In general, the hydrolysis of membrane phospholipids by PLA_2_s generates a large number of pro-inflammatory lipid mediators, including prostaglandins, prostacyclins, thromboxanes, and leukotrienes. This “inflammatory soup” changes the chemical environment of the nerve fibers resulting in peripheral sensitization. These sensitizing compounds are recognized by receptors expressed in the nociceptive neurons, for example, G-protein coupled receptors (GPCR), tyrosine kinase receptors, and ionotropic receptors whose activation contribute to heightening the nerve fiber sensitivity to temperature or touch, in other words, to hyperalgesia [39].

Inflammation frequently occurs in the periphery at the site of injury. The sustained activation of C and Aδ nociceptors by inflammatory mediators (or other stimuli) induces transcriptional and post-translational modifications, including upregulation of Na^+^ channels and protein kinase A and C activation with subsequent Ca^2+^ and Na^+^ channels sensitization. At the spinal cord, inflammation induces pre-synaptic neurotransmitters (glutamate, calcitonin-gene related peptide, substance P) and ATP release, therefore potentiating the activation of secondary neurons and/or stimulating postsynaptic plasticity. This neuroplasticity includes “unsilencing” of glutamatergic NMDA receptors and the upregulation of glutamatergic AMPA receptors. The central sensitization also involves the release of modulatory substances, including cytokines and chemokines from neurons and glia cells (astrocytes/microglia), as well as loss of inhibitory input (descending pain inhibitory circuits) [41].

Most anti-inflammatory and analgesics can relieve pain by decreasing the prostanoids or acting in the descending pain modulatory system. The non-steroidal anti-inflammatory drugs most commonly control prostaglandin synthesis by inhibiting cyclooxygenases (Cox-1 and Cox-2), for example, ibuprofen and aspirin. Conversely, drugs that act in the descending pain modulatory circuits decrease the nociceptive input in the central nervous system by releasing neurotransmitters that can exert an inhibitory action, such as opioids, acetylcholine [42], noradrenalin [43], and serotonin [43,44,45]. These inhibitory pathways act at physiological states, controlling the positive stimuli of the nociceptive pathways, being, therefore, a pain relief target for drugs and toxins during pathological pain conditions.

As mentioned before, venoms are a rich source of PLA_2_ that contribute to the clinical signs that were observed in human envenomation [38,46,47]. Studies performed in rodents have demonstrated that sPLA_2_s isolated from the *Bothrops asper* snake venom induce hyperalgesia and this effect is mediated by biogenic amines, bradykinin, cytokines, prostaglandins, and sympathomimetic amines that may interact and be sequentially released [48]. Moreover, the Lys49-PLA_2_ isolated from *Bothrops* venoms induces ATP and K^+^ release from muscle cells that can directly induce pain by activating purinergic receptors or inducing membrane depolarization of peripheral sensory nerves [49,50].

Studies have demonstrated that IB, IIC, V sPLA_2_, as well as IVA cPLA_2_, and VI iPLA_2_ are constitutively expressed in the nociceptive pathways, such as the spinal cord [51,52]. Of note, IVA cPLA_2_ and VI iPLA_2_ groups are highly expressed in this tissue. Despite the fact that peripheral inflammation does not change spinal expression of PLA_2_, intrathecal injection of sPLA_2_ inhibitors (LY311727) or cPLA_2_ inhibitors (knockdown antisense oligonucleotide, AACOCF3, MAFP, or dexamethasone) blocks nociceptive behavior in established experimental models of hyperalgesia (formalin and carrageenan) by decreasing the PGE_2_ tissue levels [52,53,54]. Conversely, iPLA_2_ inhibition does not interfere with the nociceptive behavior. Taken together, these data reinforce that sPLA_2_ and cPLA_2_ participate in the facilitation of spinal pain processing through modulation of PGE_2_ synthesis. PGE_2_ are also critical in the disinhibition of the inhibitory pain system, as it selectively blocks inhibitory glycinergic neurotransmission onto superficial dorsal horn neurons by a mechanism involving the activation of EP_2_ receptors [55].

Studies seeking the development of highly potent and selective PLA_2_ inhibitors could contribute to the development of novel therapeutic agents for the treatment of inflammatory pain, including arthritis and envenomation-induced pain. In this regard, oral administration of cPLA_2_ inhibitor (arachidonyl trifluoromethyl ketone) alleviates pain that is induced by cauda equina compression in rats [56]. Kokotou and collaborators [57] have developed a selective IVA cPLA_2_ inhibitor. However, these in vitro findings still have to be validated in preclinical studies. Interestingly, varespladib and its orally bioavailable prodrug, methyl-varespladib have high-level sPLA_2_ inhibition against 28 snake venoms. In vivo studies with varespladib showed that this inhibitor protects against lethal doses of the *Micrurus fulvius* and *Vipera berus* snake venoms, and suppressed venom-induced sPLA_2_ activity in rats that were challenged with 100% lethal dose of *M. fulvius* venom [58]. Despite these positive results concerning lethality, studies on inhibitors (or mixture of inhibitors) efficacy in the prevention of venom-induced local tissue damage, such as inflammation and pain, are still necessary.

## 4. Crotoxin, a Heterodimeric Neurotoxin from *Crotalus durissus terrificus* Venom That Induces Analgesia

Crotoxin (CTX) is the main toxic component in the venoms of South American *Crotalus durissus terrificus* rattlesnakes. This toxin is a neurotoxin that exerts lethal action through a potent blockade of neuromuscular transmission, mostly at the presynaptic level, preventing acetylcholine release from peripheral neurons at the neuromuscular junction [59,60]. Although neuromuscular blockade by presynaptic activity has been the most studied effect of CTX, cardiotoxic, nephrotoxic, and myotoxic activities have also been observed [11,61,62]. Furthermore, several studies have shown that crotoxin inhibits acute and chronic pain [63,64,65].

CTX is a heterodimeric complex consisting of a non-covalent association between an acidic non-enzymatic protein (crotoxin A, CA or crotapotin) and a basic toxic phospholipase A_2_ (crotoxin B or CB) [66,67]. CA is formed by three polypeptide chains (α, β and γ, where the α and β chains are α-helices with loops at the terminal positions, and the γ chain is a disordered loop) linked by disulfide bonds, whereas CB is a class II phospholipase A_2_ [66,68]. CA is generated from a PLA_2_-like precursor (pro-CA) by the removal of three peptides, leaving unchanged the molecule core linked by disulfide bonds [69]. CA does not display any toxicity itself, but it decreases the enzymatic activity, at the same time that it enhances the pharmacological activity of CB [67,70,71,72]. Despite its higher catalytic activity, isolated CB has a weaker toxic action on skeletal muscle and on neuromuscular junctions, as compared to CTX. The presence of CA strongly potentiates muscle necrosis and the blocking of neuromuscular transmission, as well as enhances the inhibition of acetylcholine release induced by CB [70,71,72]. Apparently, the role of CA is to prevent CB adsorption to non-saturable binding sites, thereby restricting its binding to critical target sites at neuromuscular junctions [11]. In fact, it was recently demonstrated that titration of CA in CB tetramers causes the dissociation of CB oligomers, restoring the CTX heterodimer [68]. Furthermore, the inhibition of the catalytic activity of CB, as well as the enhancement in CB pharmacological activity caused by CA, suggest that other regions of CB, besides the active site, may be involved in its mechanism of action.

Some studies have suggested that the N- and C-terminal regions of CB are involved in the neurotoxic activity of CTX. This suggestion is based on data showing that antibodies against the C-terminal part of AtxA, a neurotoxin from the *Vipera ammodytes ammodytes* snake, bind to the C-terminal peptides of CB, protecting mice against the lethal effect potency of CB [73]. In turn, peptide-array analysis showed that the N-terminal region of CB (Phe11-Ala18) could constitute a pharmacological site of this protein [74], and chemical modification of Y22 reduced CB neurotoxicity and its binding affinity for presynaptic membranes [75]. Moreover, the predicted i-face of CB includes several residues from the N-terminal region [76,77]. Some studies have also suggested that neurotoxicity of CTX depends on its interaction with a protein acceptor on presynaptic membranes [78]. Other studies have showed that presynaptic toxins from snake venoms, in general, can enter into the lumen of synaptic vesicles following endocytosis and hydrolyze phospholipids from the inner leaflet of the membrane [79]. Remarkably, it was demonstrated that the CB subunit can be internalized in cerebrocortical synaptosomes, independently of the presence of CA or of its catalytic activity [80]. Finally, it was recently demonstrated that CTX can interact with nicotinic acetylcholine receptors [81] and that the CB subunit can interact with, and be an allosteric modulator of, cystic fibrosis transmembrane regulator (CTRF) chloride channel [82]. It was also demonstrated that CB is able to interact with prokaryotic proton-gated ion channel GLIC, a bacterial homolog of pentameric ligand-gated ion channels [83].

Several different CA and CB isoforms have been isolated and characterized. The random association of different isoforms of both CA and CB may result in at least sixteen distinct CTX complexes, which can possibly coexist in a single specimen of *C. d. terrificus* snake [71,84]. Different isoforms of CB exhibit slight modifications in the enzymatic and pharmacological properties of the CTX. CTX isoforms are grouped in classes I and II [71]. When complexed to CA, the CB_b_, CB_c_ and CB_d_ isoforms (class I isoforms) are more toxic, have less enzymatic activity, and dissociate from CA more slowly than the CBa2 isoform (class II isoforms) [71].

Despite being the first animal toxin crystallized, in 1938 [85], only very recently the first structural data that provided insights into CTX mechanism of action, at molecular level, was available. In 2009, the crystal structure of a tetrameric complex was described, being formed by two dimers of CB_c_-CB_a2_ isoforms (class I and class II isoforms, respectively) [86]. The high concentration of polar residues at CB surface proteins induces its oligomerization by formation of a well-structured net of hydrogen bonds (17) and salt bridges (6) between the monomers, to form dimers and tetramers of this subunit [86,87]. Later, in 2011, a class I isoform of CTX (CA_2_-CB_b_ isoform) was reported, highlighting the role, in the CA/CB interface, of the residues Trp36 from CA and Trp31 and Trp70 from CB [66]. However, in this structural model, Trp36 from CA and Trp70 from CB are partially exposed and Trp90 from CB is totally exposed to the solvent. Steady-state and time-resolved spectroscopy fluorescence data have suggested that tryptophan residues become hidden in CA/CB interface in the CTX heterodimer structure [68]. In this context, a new structural model of CTX structure was recently reported, by combining spectroscopy fluorescence and small angle X-ray scattering data (SAXS) [68]. In this model, Trp31 from CA and Trp36 and Trp70 from CB are completely buried in the inner CTX structure; and, Trp90 from CB is partially occluded by N-terminal region of β-chain of CA [68]. In addition, the CTX SAXS model revealed some flexible regions of CA that were not modeled in CTX crystal structure, due to the absence of electron density, thus providing an overall representation of CA and CTX tridimensional structures [68]. Moreover, based on the analysis of differences in the position of N and C-terminal regions of CB, at both of the structural models, a hypothesis on CTX mechanism of action was proposed.

**Figure 3:**
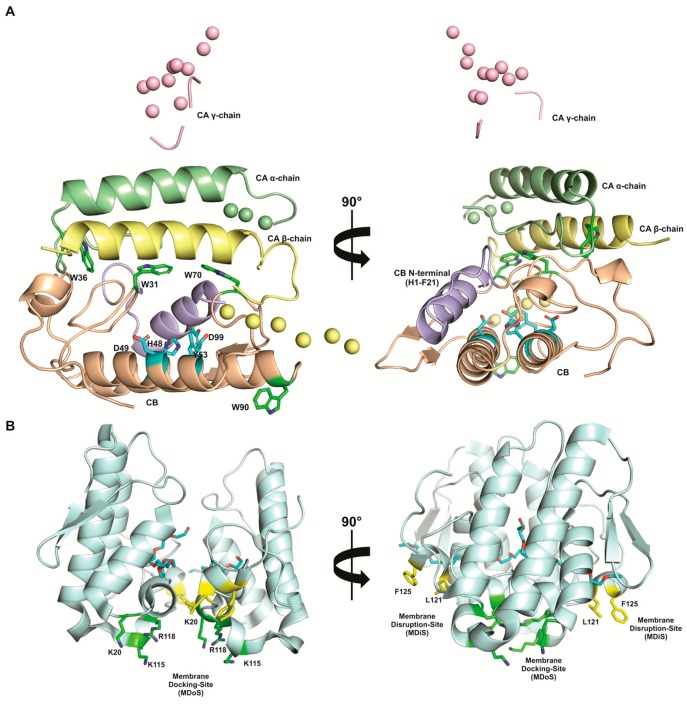
Cartoon representation of the quaternary structure of (**A**) crotoxin (CTX), obtained by small angle X-ray scattering (SAXS); and (**B**) BthTX-I, a Lys49-PLA_2_ from the *Bothrops jararacussu* snake venom, as representative of PLA_2_-like proteins group, obtained by protein crystallography (PDB ID 3IQ3). In the CTX structural model (panel A), the two subunits that form the heterodimer; crotoxin B (CB; in light brown) and crotoxin A (CA) are shown. The α, β and γ polypeptides chains that constitute the structure of CA are highlighted in green, yellow and pink, respectively. The flexible loops that are not present in the CTX crystal structure (PDB ID 3R0L; [43]), but were modelled in CTX SAXS model as dummy residues [45], are showed as spheres. The tryptophan residues of CTX (W36 from α-chain of CA; W31, W70 and W90 from CB) and the catalytic network of CB (His48, Asp49; Tyr53 and Asp99) are highlighted in green and cyan sticks, respectively. The N-terminal exposed to the solvent of CB in CTX heterodimer is highlighted in blue. In the Lys49-PLA_2_ structural model (panel B), the most important structural aspects of these proteins are highlighted: the hydrophobic molecule in hydrophobic channel that causes the activation of the protein (a polyethylene glycol molecule, showed in cyan sticks); the Membrane Docking-Site (MDoS) formed by Lys20; Lys115; and, Arg118 residues from both monomers and responsible for the stabilization of the protein on the target membrane (green sticks); and the Membrane Disruption-Site (MDiS), formed by Leu121 and Phe125 from both monomers, that penetrates in the target membrane causing a disorganization of the lipid bilayer, allowing an uncontrolled influx of ions (i.e., Ca^2+^ and Na^+^), and, consequently, cell death. Modified from Fernandes et al., 2017: Scientific Reports, volume 7, page 43885, Spring Nature, copyright 2017 [68].

As previously discussed, taking into account that CA enhances the CB toxicity at the neuromuscular junction, by the formation of CTX heterodimer, a region of CB that is not in contact with CA, but is exposed to the solvent, may be able to interact with the membrane target. A structural analysis of N- and C-terminal regions of CB in CTX crystal structure, showed that both regions are completely buried in the CA/CB interface; however, in the CTX SAXS structural model, the N-terminal region (His1-Phe21) is not in the CA/CB interface and it is exposed to the solvent (Figure 3a). This observation leads to the hypothesis that the N-terminal region of CB, that contains several residues from the predicted i-face of CB, would be the first binding site of CTX at the membrane target sites. After CB N-terminal binding to the target, CA would dissociate from CB, allowing for the interaction of the C-terminal of CB with the target and making the catalytic site of CB totally accessible [68].

## 5. Antinociceptive Effect of Crotoxin

Besides the widely described toxic effects of crotoxin, several experimental data have demonstrated that this toxin induces analgesic effect observed both in animals and humans.

The antinociceptive effect of crotoxin has been evidenced in different behavioral models of pain, being this effect centrally mediated. In this regard, Zhang et al. (2006) [64] demonstrated that the toxin, injected in mice and rats, in the periphery (i.p. route) or in the CNS (intracerebral ventricular or periaqueductal gray area injections) induces antinociception. The observed effect was not due to a possible impairment on motor activity, as CTX has no effect on the spontaneous mobility of animals, as well as it did not involve muscarinic or opioid receptors, because the inhibition of receptor activation does not interfere with the antinociceptive effect. Nogueira-Neto et al. (2008) [65], evaluating the antinociceptive effect of CTX in a model of neuropathic pain that was induced by rat sciatic nerve transection, observed that the immersion of proximal and distal nerve stumps in a CTX solution (0.01 mM for 10 s), immediately after nerve transection, prevented the development of neuropathic pain. This was a long-lasting antinociceptive effect, as it was detected 2 h after the local application of the toxin and persisted for 64 days thereafter. This effect involves the participation of central muscarinic receptors and is partially mediated by the activation of alpha-adrenoceptors and 5-HT receptors. Eicosanoids that were derived from the lipoxygenase pathway also modulate the crotoxin effect. In addition to the analgesic effect, these authors demonstrated that CTX also delayed, but did not avoid, neurectomy-induced neuroma formation.

The effect of CTX on the activity of neurons from the thalamic parafascicular nucleus (Pf), an important relay in the ascending nociceptive pathways, was also investigated. In this study, the electrical discharge of Pf neurons was electrophysiologically recorded. The results demonstrated that intracerebroventricular injection of CTX inhibited pain-evoked unit discharge of these neurons. This effect was not altered by muscarinic and opioid receptor antagonists [63]. Additional studies using blood oxygen level dependent functional Magnetic Resonance Imaging (BOLD fMRI) analysis [88], confirmed the participation of specific areas of the CNS in the antinociceptive effect of crotoxin, through the demonstration of BOLD signal reduction caused by the toxin, not only in brain input structures, but also in higher order processing structures, like primary and secondary somatosensory cortices, which are relevant for pain perception.

It is well demonstrated that crotoxin displays immunomodulatory and anti-inflammatory actions [89]. Therefore, despite the central mediation of the analgesic effect of crotoxin, a contribution from the anti-inflammatory and immunomodulatory actions of the toxin to its analgesic effect should be considered.

The effect of crotoxin on cancer pain has also been evaluated. Brigatte et al. (2016) [90] demonstrated in a rat Walker 256 tumor growth model, which five consecutive injections (1 s.c. injection/day) of crotoxin reduces tumor growth and new blood vessel formation by a mechanism involving lipoxin A_4_. These authors also showed that crotoxin inhibits mechanical hyperalgesia and low-threshold mechanical allodynia that is caused by the tumor cells (unpublished data). Opioid mechanisms were not involved in the antinociceptive effect of the toxin, as it was not modified by naloxone administration.

The effect of crotoxin on cancer was also evaluated in human beings. A Phase I clinical trial was performed on patients with solid tumors that were refractory to conventional therapy. Even during the phase I clinical trial, which was designed to establish the dose of toxin for the next clinical trial phase and the parameters for the pharmacokinetics study, some patients presented a reduction of tumor mass (more than 50%) and also the significant inhibition or even disappearance of pain symptoms [91]. Costa et al. 2001 [92] evaluated in a Phase I clinical trial, the anti-tumoral effect of a compound named VRCTC-310-Onco, made up of crotoxin combined with cardiotoxin (from *Naja naja atra*), at equimolar ratio. The addition of cardiotoxin dissociates cytotoxicity (required for antineoplastic activity) and neurotoxicity (otherwise, its main side effect) of crotoxin and allows for a useful concentration to be achieved in vivo. During these trials, an increase in the plasma level of IL-1 receptor antagonist (IL-1ra), an endogenous antagonist of IL-1 that presents anti-inflammatory action, was detected.

The possible antinociceptive effect of crotoxin has also been evaluated in experimental models of neurodegenerative diseases. Recent data (Bernardes N. and Picolo G., unpublished data) have demonstrated that this toxin inhibits hyperalgesia in mice, in the Mog_35-55_-induced Experimental Autoimmune Encephalomyelitis (EAE) model, a valuable animal model for the study of multiple sclerosis. In this model, nociception, an important marker of the disease, is detected around one week before the clinical manifestation of motor impairment. Using the EAE model, these authors observed that crotoxin induces antinociception and also immunomodulatory effects. Pharmacological studies showed that the antinociceptive effect of the toxin, in this model, is mediated by activation of muscarinic, adrenergic, and formyl peptide receptors.

## 6. Antinociception Induced by Other Animal Venoms sPLA_2_s

### 6.1. Snake Venoms

The antinociceptive effect of other snake venom-derived phospholipases A_2_ has been also evaluated in experimental studies. Dyachenko et al. (2013) [93] investigated, in mice, the antinociceptive effect of two neurotoxic phospholipases A_2_ (HDP-1 and HDP-2) that were isolated from *Vipera nikolskii* venom. Both phospholipases were heterodimers that were made up of two non-covalently bound subunits, being each heterodimer composed of an enzymatically active basic subunit and an inactive acidic subunit. In this study, the HDP-2 increased the hot plate latencies, but this effect was only observed at the maximal tolerated dose. When considering that at this dose the animals presented severe symptoms of intoxication, including depression, a marked decrease in locomotor activity and breath rate, among other symptoms, the observed effect on hot plate test might be a consequence of general intoxication rather than a specific decrease in pain sensitivity.

### 6.2. Bee Venom

Bee venoms have been used as analgesics for centuries, especially through a technique named apipuncture. This technique consists in the application of bee venom in specific acupoints, as a part of Oriental Medicine [94]. Their analgesic effects have been attributed to activation of α_2_-adrenergic and/or serotonergic receptors [95,96,97,98]. In addition to the whole venom, some phospholipases A_2_ isolated from the venom have been studied as analgesics. Li et al. (2015) [99] demonstrated the analgesic effect of a bee venom-derived phospholipase A_2_ (bvPLA_2_) in a model of neuropathic pain. In this study, neuropathic pain was induced in mice, by a single infusion of oxaliplatin, a compound that is widely used to treat metastatic colorectal cancer. The bvPLA_2_ treatment markedly inhibited oxaliplatin-induced acute cold and mechanical allodynia. Antinociception that is induced by the phospholipase A_2_ involves the activation of the noradrenergic system, via α_2_-adrenegic receptors. In contrast to what is observed for the whole venom, the serotoninergic system is not involved in the antinociceptive effect induced by the sPLA_2_. These results indicate that bvPLA_2_ contributes, at least partially, to the antinociceptive effect of the bee venom. It was also observed that bvPLA_2_ suppresses the oxaliplatin-induced macrophage infiltration and the increase in IL-1β level in the DRG [99].

## 7. Animal Venom-Derived Inhibitors of Phospholipases A_2_ as Analgesics

Compounds isolated from animal venoms could also interfere with pain and inflammation by inhibiting endogenous or exogenous phospholipases A_2_ activity. Two compounds that are isolated from the sponges of the *Luffariella* family (manoalide and luffariellolide) and another one, from the sponge *Cacospongia mollior* (scalaradial), display analgesic effect due to their anti-inflammatory properties. These three compounds have either latent aldehyde groups, CH=O (the lactols, C-OH) or actual aldehyde groups, which react with one or more lysine residues of phospholipases A_2_, forming Schiff-base (imine) linkages, being able to inhibit phospholipase activity [100].

Table 1 summarizes the main data on the antinociceptive effects of snake venom sPLA_2_s or PLA_2_ inhibitors.

## 8. Nociceptive Effects of Animal Venom sPLA_2_s

Inflammation and pain, particularly at the site of injection, are important symptoms of human envenomation by poisonous animals [38,46,47]. Animal venoms consist of a rich and complex mixture of components that include enzymes, protein, and peptide toxins, which display a variety of biological functions through the action in different molecular targets [102,103,104]. Among these components, the secretory phospholipases A_2_ (sPLA_2_s) are abundant in animal venoms, especially in Elapidae and Viperidae snake venoms. Those sPLA_2_s display important myotoxic and neurotoxic activities, being considered relevant players in the generation of inflammation and pain that is observed in humans and animals injected with these venoms [38,46,47]. Due to the clinical relevance of animal venom-induced inflammation, and taking into account that serum therapy is not efficient in controlling the local effects (including inflammation and pain) that are caused by poisonous animals, experimental studies have been carried out in order to elucidate the mechanisms involved in those phenomena and to characterize the venom components that contribute to them. As sPLA_2_s are important components of animal venoms, and, based on the well-demonstrated inflammatory and painful role of secretory phospholipases A_2_ and their involvement in different human diseases, efforts have been made to understand the role of sPLA_2_s in pain and inflammation that is induced by animal venoms [38,47,105].

In this regard, *Bothrops* snake venoms have been widely studied, due to the pronounced inflammatory response and tissue injury observed at the site of venom injection [106,107,108,109,110,111,112]. *Bothrops jararaca* and *Bothrops asper* snakes are responsible for the majority of the accidents caused by snakes in Brazil and Central America, respectively [109,113]. Data from the literature have shown that inflammation and pain caused by the venoms of *B. jararaca* and *B. asper* are multi-mediated processes, involving the participation of an array of inflammatory mediators, such as histamine, 5-hydroxytryptamine, bradykinin, and lipid-derived mediators (prostaglandins, leukotrienes, and PAF), as well as the participation of leukocyte cells [114,115,116,117,118,119,120,121]. Interestingly, studies have indicated that distinct mechanisms are involved in the development of hyperalgesia and edema induced by *Bothrops* venoms, being hyperalgesia more readily controlled by drugs endowed with anti-inflammatory activity [120]. As PLA_2_s comprise 15–25% of *Bothrops asper* venom proteins [109], Chacur et al. (2003) [48] first investigated the contribution of secretory phospholipases A_2_ toxins to nociception induced by *B. asper* venom. These authors showed that Myotoxin II (MT-II), a Lys-49 PLA_2_ devoid of enzymatic activity, and Myotoxin III (MT-III), an Asp-49 PLA_2_, that was isolated from this venom, when injected into the rat hind paw, cause local mechanical hyperalgesia of rapid onset and similar time-course. In contrast, only the catalytically active enzyme MT-III, induces allodynia, indicating that enzymatic activity, although not being essential for the generation of pain, is important to determine the intensity of the nociceptive phenomenon. Studies on the mechanisms involved in the pain-enhancing effects caused by those myotoxins have indicated that hyperalgesia induced by the Lys-49-PLA_2_ results from the action of several mediators that may be sequentially released, and/or may interact for the induction of the nociceptive phenomenon. These mediators include histamine and serotonin (partially involved), suggesting the participation of mast cells in this phenomenon; sympathomimetic amines; bradykinin; cytokines (TNFα and IL-1); and, prostaglandins. As the Lys-49 PLA_2_ (MT-II) is devoid of enzymatic activity, it cannot be involved, per se, in the release of arachidonic acid. Therefore, the synthesis of prostaglandins by MT-II may result from the action of another mediator that is released by the myotoxin. One such candidate is bradykinin (BK). It is well demonstrated that BK, acting on B2 receptors in nociceptors, is an important mediator of pain. Bradykinin can directly stimulate nociceptive neurons and also sensitize nociceptors to other stimuli, through the release of cytokines and the generation of arachidonic acid and prostanoids [122,123,124,125,126]. On the other hand, prostanoids are not involved in the hyperalgesic action of the catalytically active Asp-49 PLA_2_ (MT-III). For the MT-III, the studies have shown that bradykinin plays a major role in the generation of hyperalgesia [48]. Studies on the central mechanisms that are involved in hyperalgesia and allodynia induced by both phospholipases A_2_ (MT-II and MT-III) suggest that nitric oxide and prostanoids, released in the spinal cord, are involved in the pain-enhancing effects of the sPLA_2_s toxins [127]. Furthermore, activation of glial cells (astrocytes and microglia) in the dorsal horn of the spinal cord, also contribute to the nociceptive phenomena caused by these phospholipases A_2_ [127]. However, the time course of activation of these cells seems to depend on the presence of the catalytic activity, because the activation of astrocytes and microglia is detected by 1 h after the injection of the catalytically active MT-III, whereas marked activation of glia by the catalytically inactive MT-II was only detected 8 h after treatment. Activation of glial cells by the venom’s sPLA_2_s could contribute for the pain enhancing effects that are caused by these toxins. This suggestion is based on experimental evidence, indicating that central sensitization is an important phenomenon for the development of hyperalgesia and allodynia. As mentioned before, central sensitization is not uniquely governed by neuronal communication, but also depends on activation of spinal glial cells, the release of cytokines and chemokines in the spinal cord, and a cross-talk between neurons and glial cells [128,129,130]. Taken together, the data herein described strongly suggest that both myotoxic PLA_2_s play a significant role in hyperalgesia induced by *Bothrops asper*’s whole venom.

Based on the fact that during *Bothrops* envenomation, venom is frequently delivered intramuscularly [109], being potentially able to reach peripheral nerve bundles, Chacur et al. (2004) [131] evaluated, for the first time, the hyperalgesic effect of *Bothrops asper* MT-II and MT-III toxins injected around rat healthy sciatic nerve. In contrast to the data observed after intraplantar injection, both of the myotoxins induced mechanical allodynia. Pharmacological assays showed that this phenomenon is mediated, in the spinal cord, by the activation of glial cells ipsilateral to perisciatic sPLA_2_s administration, and by the release of nitric oxide, IL-1 and IL-6. It is important to point out that nitric oxide and IL-1 are not involved in the allodynic effect that is induced by the catalytically active Asp-49 PLA_2_ when subcutaneously injected. The difference in the results obtained in the studies of Chacur et al. [127,131] suggests that the local of toxins delivery may influence the severity of the pain symptom during envenomation by poisonous animals.

The possible involvement of a phospholipase A_2_ toxin in hyperalgesia induced by the venom of *Bothrops moojeni*, a snake responsible for most of the snakebites in the central region of Brazil [132], was suggested by Mamede et al. (2016) [133]. These authors demonstrated, using behavioral models of pain evaluation, that bradykinin is an important mediator of the pain enhancing effect that is induced by *B. moojeni*, and that the venom’s PLA_2_ activity contributes to this phenomenon. In an attempt to further characterize the molecular mechanisms that are involved in the action of sPLA_2_s from *Bothrops* venoms, and based on the ability of these myotoxins, including Lys-49 myotoxins, to release ATP from skeletal muscle [49], the involvement of purinergic signaling in the nociceptive effects of these sPLA_2_ has been investigated. In an elegant experimental work, Zhang et al. (2017) [134] demonstrated that the purinergic signaling contributes to nociception induced by BomoTx, a toxin that is closely related to Lys-49 myotoxins, isolated from *Bothrops moojeni* (Brazilian lancehead pit viper) venom. In their work, Zhang et al. (2017) demonstrated, using pharmacological and electrophysiological assays, that BomoTx: (a) is a nociceptive Lys49 myotoxin, able to induce non-neurogenic inflammatory pain, thermal hyperalgesia, and mechanical allodynia; (b) induces ATP release from neurons, being the pannexin hemichannels downstream mediators of the toxin-evoked ATP release. Subsequent activation of P2X receptors is observed on transiently responding neighbor’s cells. These authors also demonstrated that nonneurogenic inflammation and thermal hypernociception are mediated mainly through TRPV1-positive fibers; whereas, mechanical allodynia requires purinergic signaling through TRPV1-negative, P2X2-, and/or P2X3-positive neurons (Aδ-fibers). These data further support the involvement of sPLA_2_s toxins in pain that is induced by *Bothrops* venoms, as well as the contribution of purinergic signaling to pain, evidencing the potential therapeutic use of antipurinergic drugs.

As mentioned earlier in this review, experimental data have indicated that the presence of the catalytic activity in the sPLA_2_ myotoxin is not essential for its nociceptive activity. Concerning the structural basis for the action of the Lys-49 PLA_2,_ devoid of enzymatic activity, the C-terminal cationic/hydrophobic sequence 115–129 of the molecule is involved in the generation of hyperalgesia by the toxin. This suggestion is based on the observation that intraplantar injection of a synthetic peptide corresponding to the C-terminal sequence 115–129 of MT-II, the Lys-49 PLA_2_s from *B. asper* venom, caused hyperalgesia of similar time course, but varying magnitude, than that induced by the native protein. It has been shown that the C-terminal sequence 115–129 is also responsible for the cytolytic and edematogenic activity of this Lys-49 PLA_2_s [135,136]. In contrast, a homologous peptide that was derived from the Asp-49 PLA_2_ myotoxin (*B. asper* MT-III) did not show any nociceptive effect [48]. Studies using the Asp-49 PLA_2_, then chemically modified byp-bromophenacyl bromide, indicate that for this myotoxin, the enzymatic activity is important for the generation of the nociceptive effect [48].

In order to characterize the amino acid sequences in the C-terminal cationic/hydrophobic sequence of myotoxic Lys-49 PLA_2_s, which are responsible for the nociceptive effect, Zambelli et al. (2017) [137] carried out assays using scanning alanine mutagenesis in the active-site and C-terminal region of BthTx-I, a Lys-49 PLA_2_ from the venom of *Bothrops jararacussu*. Scanning alanine mutagenesis has been shown to be a useful strategy to study the structural determinants of the activities of Lys-49 PLA_2_. In this sense, Chioato et al. (2002) [138] have demonstrated, for the BthTx-I, that the Arg115Ala and Arg116Ala mutants do not display membrane-damaging activities, whereas the Lys122Ala mutant lacks myotoxic activity. In addition, His48Gln substitution, which eliminates any possible catalytic activity, does not interfere with the membrane damaging properties of the toxin. Using these same BthTx-I mutants, Zambelli et al. (2017) [137] showed, for the first time, that distinct residues are involved in hyperalgesia and edema induced by BthTx-I. These authors also demonstrated that the cytolytic activity is essential for the hyperalgesic effect, but not for edematogenic activity, as the mutations K115A and K116A abolished hyperalgesia without interfering with edema. These data corroborate Chacur et al. (2003) [48] data showing that edema and hyperalgesia can occur in a non-dependent manner. Furthermore, the amino acids arginine at position 118 and lysine at position 122 seem to be important for the biological activity of the BthTx-I PLA_2_, as the mutants did not induce nociception. The results obtained with the mutants PLA_2_ also indicate that hyperalgesia induced by BthTx-I depends on the main biological activities of the toxin (see section “Lys49-PLA_2_s, a PLA_2_-like proteins subgroup that induces hyperalgesia in a catalytic activity-independent way” below, for more detailed data on the mechanism of action of PLA_2_-like proteins). Taken together, these data contribute to the understanding of the structural determinants of pain-inducing effects of venoms phospholipases A_2_ and to the future characterization of molecular targets for PLA_2_-induced pain.

Based on their biological actions, secretory phospholipases A_2_ isolated from animal venoms have been considered valuable tools to study the molecular mechanisms that are involved in human inflammatory diseases, including pain, in which secretory phospholipases A_2_ are relevant for the pathophysiology of the disease.

Severe abdominal pain is a common and important clinical symptom in patients with acute pancreatitis [139], which is difficult to control and to experimentally assess. Therefore, standardization of models that mimic this condition and that allow for the characterization of possible targets for pain control is an unmet need.

Several lines of evidence have indicated that secretory PLA_2_s display an important role in pancreatitis. Also, inhibitors of these enzymes have been evaluated in experimental models for the treatment of this disease [140]. Studies have also indicated that the levels of sPLA_2_s, mainly group II secretory PLA_2,_ in the serum of patients with pancreatitis are well correlated with the severity of disease [140]. Based on these data, Camargo et al., in 2005 [140], used, in a pioneer study, sPLA_2_s, variants Lys-49 and Asp-49, which were isolated from snake venoms as a tool for the development of an experimental model of pancreatitis. In this study, the authors injected the sPLA_2_s into the common bile duct of rats, to induce acute pancreatitis. The snake sPLA_2_s caused alterations in the pancreas and also the lungs, which mimic those that were observed in patients with acute pancreatitis. These authors also showed that the presence of catalytic activity in the sPLA_2_ is not essential for the observed pancreatic inflammatory response; however pulmonary inflammation depends, at least partly, on the sPLA_2_ catalytic activity. Using a neurotoxic sPLA_2_ isolated from the South American rattlesnake *Crotaluss durissus terrificus* venom, Camargo et al. (2011) [141] observed, in addition to pancreas inflammation (edema and neutrophil infiltration) and increased serum amylase, the presence of acute abdominal hyperalgesia. These authors also evidenced that the NK1 receptors are involved in the early abdominal hyperalgesia, indicating a possible role of these receptors in the generation of pain in human pancreatitis condition. Despite the presence of abdominal hyperalgesia, which indicates the presence of sensitization, the crotalid sPLA_2_ do not directly depolarize sensory fibers. Therefore, sensitization must result from the action of other components that are able to sensitize nociceptors, such as bradykinin, which was shown to mediate pancreatic inflammation that is induced by snake’s sPLA_2_s [142]. Taken together, these data indicate that sPLA_2_s isolated from snake venoms can be used to mimic the main clinical findings of human pancreatitis, fostering the understanding of the mechanisms that are involved in the development of this disease.

In contrast to the results on the inflammatory action of the sPLA_2_ isolated form *C. d. terrificus* venom, as described above, experimental data have suggested that this neurotoxic PLA_2_ displays anti-inflammatory effects [89]. This sPLA_2_ is a component of the main toxin of the crotalid venom, named crotoxin. Several experimental works have indicated that crotoxin exerts immunomodulatory, anti-inflammatory, and analgesic (discussed in detail in the present review) actions [89]. In these studies, the effects of the whole toxin (crotoxin) were evaluated. On the other hand, in the studies that were carried out by Camargo et al. (2011), the purified sPLA_2_ was used for the induction of pancreatitis and abdominal hyperalgesia. As pointed out in Introduction, crotoxin is a heterodimeric complex, consisting of a noncovalent association between an acidic non-enzymatic protein (crotoxin A, CA, or crotapotin) and a basic toxic phospholipase A_2_ (crotoxin B or CB) [66,67]. Despite its higher catalytic activity, isolated CB (PLA_2_) has a weaker toxic action as compared to CTX. CA does not display any toxicity itself, but decreases the enzymatic activity, and, at the same time, enhances the toxic activity of CB [67,70,71,72]. Therefore, when using the purified CB component (PLA_2_), as in the study of Camargo and collaborators, the increased enzymatic activity could be responsible for the observed inflammatory effects.

Another study on the use of venom secretory PLA_2_s as tools for the standardization of experimental models that mimic human inflammatory diseases, was carried out recently, by Dias et al. (2017) [143]. In this study, the authors, using a sPLA_2_ that was isolated from a *Bothrops* snake venom, standardize an experimental model of arthritis.

Joint diseases, such as rheumatoid arthritis and osteoarthritis, can cause functional disability, interfering with the patient’s quality of life. Arthritis symptoms include aching, stiffness, swelling, and pain in the affected joint [144,145]. Effective and/or protective treatments for these pathologies are still a challenge [146,147]. Therefore, the standardization of animal models that share the same characteristics of human arthritis are important for the characterization of new molecular targets and for new drug development, aiming at the adequate control of the disease.

Articular inflammation is a multi-mediated event, involving the participation of phospholipases A_2_ [148]. In order to further characterize the role of sPLA_2_ in acute joint arthritis, Dias et al. (2017) [143] evaluated the effect of the *B. asper* Lys-49 PLA_2_ myotoxin (MT-II) that was injected in rats into the left tibio-tarsal or femoral-tibial-patellar joints. In this study, the authors used the MT-II, which is devoid of catalytic activity, aiming to study joint inflammation without the interference of exogenous enzymatic phospholipid degradation. Intra-articular injection of the Lys-49 PLA_2_ induced plasma extravasation in the knee joints, polymorphonuclear cell influx, and hyperalgesia, sharing many of the features that were observed in human arthritis. Pharmacological assays showed that MT-II-induced hyperalgesia is a multimediated phenomenon, involving the participation of eicosanoids (through the activation of endogenous PLA_2_s), bradykinin, cytokines, and endothelin. Articular pain-enhancing effects of this myotoxin are also dependent on the cellular influx to the joint. Based on these data, it was suggested that venom’s sPLA_2_ could be considered a valuable tool for the understanding of the cellular and molecular mechanisms that are involved in arthritis, as well as for the evaluation of new therapeutic approaches.

Table 2 summarizes the main data on the pain-enhancing effects of snake venom sPLA_2_s.

## 9. Lys49-PLA_2_s, a PLA_2_-Like Proteins Subgroup That Induces Hyperalgesia in a Catalytic Activity-Independent Way

Snake venoms from *Bothrops*, *Trimesurus* and other Viperidae family genera contain a subtype of sPLA_2_s, the PLA_2_-like proteins, that have similar tertiary structures to sPLA_2_s, but do not exhibit catalytic activity due to the Y28N and D49K/S/R mutations that impair Ca^2+^ binding [20,22]. It has been demonstrated that the dimeric form of the protein is essential for the initiation of the Ca^2+^-independent membrane damaging activity [33,149]. As described before, despite being catalyticaly inactive, the sPLA_2_s-like proteins are able to induce marked local myotoxicity and other biological activities, including hyperalgesia and edema [23].

The first attempt to describe the protein region that is involved in PLA_2_-like proteins activity was carried out using a synthetic peptide (residues 115–129 of the C-terminal region) of a PLA_2_-like protein. This synthetic peptide was able to induce cytolytic activity [135]. These data led to the hypothesis that this region, formed by cationic and hydrophobic residues, may be responsible for the toxicity of these PLA_2_-like proteins. Furthermore, site-directed mutagenesis experiments on BthTX-I, a Lys49-PLA_2_ from *B. jararacussu* venom, supported the importance of the C-terminal region of the PLA_2_ myotoxic, painful, bactericidal, and damaging activities, especially the K115, Y117, R118, Y119, L121, K122, and F125 residues [138,150]. Finally, crystallographic data of nineteen PLA_2_-like protein structures from venoms of different snake species also supported the C-terminal region as one of the regions that is involved in toxic activity of these proteins, as well as pointed out the involvement of some residues of the N-terminal and hydrophobic channel regions to the biological activity of these toxins [33,151]. A mechanism of action for these proteins that fully integrates biochemical and crystallographic data was recently proposed (review in [33]). This mechanism involves protein activation towards changes in its quaternary structure and two distinct sites for interaction with cell membranes—the Cationic Membrane Docking Site (MDoS) and the Hydrophobic Membrane Disruption Site (MDiS) (Figure 3). The mechanism of action of PLA_2_-like proteins is summarized below:

The entrance of a hydrophobic molecule (e.g., fatty acid) in the hydrophobic channel of a monomer, causing the reorientation of the dimer. This reorientation approximates L121 and F125 residues and opens the hydrophobic channel of the other monomer [152];

(i) Binding of a hydrophobic molecule in the hydrophobic channel recently opened. This event characterizes the transition between inactive and active states of the protein, being the active state the dimer with a hydrophobic molecule in the hydrophobic channel of each monomer. In the active state, MDoS and MDiS regions become exposed to the solvent and aligned in the same plane with a symmetric position for both monomers [33,152];
(ii) Stabilization of the protein on the membrane by interaction of MDoS from both monomers and the phospholipid head groups on target cell membrane. MDoS is formed by K20, K115, and R118 cationic residues, but it can be aided by other positive and exposed residues, such as K80, K122, and K127 [33,149]. Indeed, several authors have shown that the cationic charge of these molecules is essential for their pharmacological properties, including hyperalgesia and inflammation [48]. Co-crystallization of inhibitors that bound to MDoS evidenced its involvement on PLA_2_s-like proteins activities [151,153];
(iii) Membrane destabilization by the penetration of MDiS from both of the monomers into the target membrane. This insertion causes a disorganization of the lipid bilayer, causing an uncontrolled influx of ions (i.e., Ca^2+^ and Na^+^), and, consequently, cell death [33,149]. MDiS is formed by L121 and P125 residues, which are conserved in the majority of PLA_2_-like proteins [33,149]. Furthermore, L and P are residues with high hydrophobic indices and membrane permeability coefficient [154,155]. Co-crystallization of inhibitors that bound to MDiS evidenced its involvement on PLA_2_s-like proteins mechanism of action [153].

## 10. Concluding Remarks

Animal venoms, particularly snake venoms, are rich sources of secreted phospholipases A_2_, which display several biological properties. These venoms can act by either inducing inflammation/pain or controlling these systems, inducing anti-inflammatory effect/analgesia. Interestingly, in some cases, a dual effect has been reported for the same phospholipase, as observed by crotoxin, where the CB subunit induces analgesia when complexed to the CA, while it causes hyperalgesia when injected independently of the complex. Based on their inflammatory and painful activities, these toxins have been widely used as tools for the understanding of the pathophysiology of inflammation and pain that is observed in human envenomation by poisonous animals, particularly snakes. These studies have revealed an important role of the sPLA_2_s from *Bothrops* species to the local inflammatory and nociceptive responses that were observed during envenomation. Also, based on the presence of sPLA_2_s in inflammatory exudates, in a variety of pathological conditions in humans, and on the structural similarity to human sPLA_2_s, secretory phospholipases A_2_ isolated from animal venoms have been successfully used for the standardization of experimental models to study the molecular mechanisms that are involved in human inflammatory diseases, including pain. The results that are obtained in these studies also support the relevance of the discovery of new sPLA_2_s inhibitors for the treatment of diseases involving PLA_2_. It is important to stress the need, in the studies using venom-isolated sPLA_2_s, of deploying additional experimental approaches, for example, “omics” approaches, to search for new molecular targets that are involved in inflammation and pain induced by sPLA_2_ toxins.

Data presented in this review also demonstrate that some venom’s sPLA_2_s, particularly neurotoxic PLA_2_s, display antinociceptive effects involving, mainly, activation of endogenous mechanisms of pain control. These toxins also display anti-inflammatory and immunomodulatory actions, which could contribute to the observed antinociceptive effect. Interestingly, sPLA_2_s that are found as a complex in the whole venom can have an opposite biological activity when uncoupled, as for crotoxin, for example. In this sense, and based on the high selectivity to their targets, animal toxins have been used as tools to uncover molecular targets that are implicated in the control of pain, and also for new analgesic drugs design. Venom’s sPLA_2_s, through their biological actions, are good candidates for the characterization of targets and signaling pathways involved in pain and its control. The studies involving these molecules are carried out using, mainly, in vivo behavioral models. To further exploit these targets and pathways, the use of in vitro models is an important and urgent need.

## Figures and Tables

**Table 1:** Animal venom-derived phospholipases A_2_ or PLA_2_ inhibitors with antinociceptive activity.

Animal	Species	Compound	Structure	Mechanism of Analgesia	Reference
Snake	*Crotalus durissus terrificus*	Crotoxin	Phospholipase A_2_	Central muscarinic receptors, α-adrenoceptors, 5-HT receptors and lypoxin A_4_ release	[63,64,65,88,90,91]
Snake	*Crotalus durissus terrificus* and *Naja naja atra*	VRCTC-310-Onco composed of crotoxin from *C. d. terrificus* and cardiotoxin from *N. n. atra*, at equimolar ratio	Phospholipase A_2_ and a sixteen amino-acidpolypeptide	Increase on the plasma level of IL-1 receptor antagonist (IL-1ra)	[92]
Snake	*Vipera nikolskii*	HDP-2	Phospholipase A_2_	Not confirmed	[93]
Bee	*Apis mellifera*	bvPLA_2_	Phospholipase A_2_	α_2_-adrenegic receptors	[99,101]
Marine Sponge	*Luffariella* family and *Cacospongia mollior*	Manoalide, luffariellolide and scalaradial	Structure containing aldehyde groups	Phospholipase A_2_-inhibitor	[100]

**Table 2:** Peripheral and Central mediators involved in pain induced by secretory phospholipases A_2_ (sPLA_2_) isolated from animal venoms.

sPLA_2_	sPLA_2_ Subtype	Venom Source	Pain-Enhancing Effects	sPLA_2_’ Structural Determinants	Mechanisms	References
Myotoxin II	Lys-49 PLA_2_	*B. asper*	Mechanical hyperalgesia (injected s.c., intra-articularly or around nerve)	C-terminal cationic/hydrophobic sequence 115–129	Periphery: histamine, serotonin, sympathomimetic amines, endothelin, bradykinin, cytokines, prostaglandins; cellular influx (intraarticular injection)	[48,143]
					Spinal cord: nitric oxide, prostanoids, IL-1 and IL-6 *, astrocytes and microglia	[48,127,131]
			Mechanical allodynia (injected around nerve)	ND ^&^	Spinal cord: nitric oxide, prostanoid,; IL-1 and IL-6 *, astrocytes and microglia	[48,127]
Myotoxin III	Asp-49 PLA_2_	*B. asper*	Mechanical hyperalgesia and allodynia (injected s.c. or around nerve)	Enzymatic activity	Periphery: Bradykinin	[48]
					Spinal cord: nitric oxide, prostanoids; IL-1 and IL-6 *, astrocytes and microglia	[48,127,131]
BomoTx	Asp-49 PLA_2_	*B. moojeni*	nonneurogenic inflammatory pain, thermal hyperalgesia, mechanical allodynia (injected s.c.)	ND ^&^	ATP release; P2X2 and P2X3 purinergic receptors activation (mechanical sensitization), involvement of TRPV1-fibers (thermal hypernociception)	[134]
BthTx-I	Lys-49 PLA_2_	*B. jararacussu*	Mechanical hyperalgesia (injected s.c.)	K115; K116; R118; K122 (in the C-terminal)	ND ^&^	[137]
CB ^#^	Asp-49 PLA_2_	*C. d. terrificus*	Abdominal hyperalgesia (injected into the common bile duct)	ND ^&^	NK1 receptors	[141]

^#^ basic toxic phospholipase A_2_ component of crotoxin, the main crotalid venom toxin; ^&^ ND: Not determined; * For the PLA_2_ administered around the nerve.

## Supplementary Materials

The following are available online at www.mdpi.com/2072-6651/9/12/406/s1. Figure S1: Two proposed catalytic mechanism for secreted PLA_2_s—the single-water and assisting-water mechanisms.
