# Supplementary material for: Secreted Phospholipases A2 from Animal Venoms in Pain and Analgesia
Source: Toxins (Basel). 2017 Dec 19;9(12):406. doi: 10.3390/toxins9120406 (PMC5744126; doi:10.3390/toxins9120406)
Supplement: Supplementary file 1 [file toxins-09-00406-s001.pdf]

Supplementary Materials: Secreted Phospholipases A2 from Animal Venoms in Pain and Analgesia

Vanessa O. Zambelli, Gisele Picolo, Carlos A. H. Fernandes, Marcos R. M. Fontes and Yara Cury

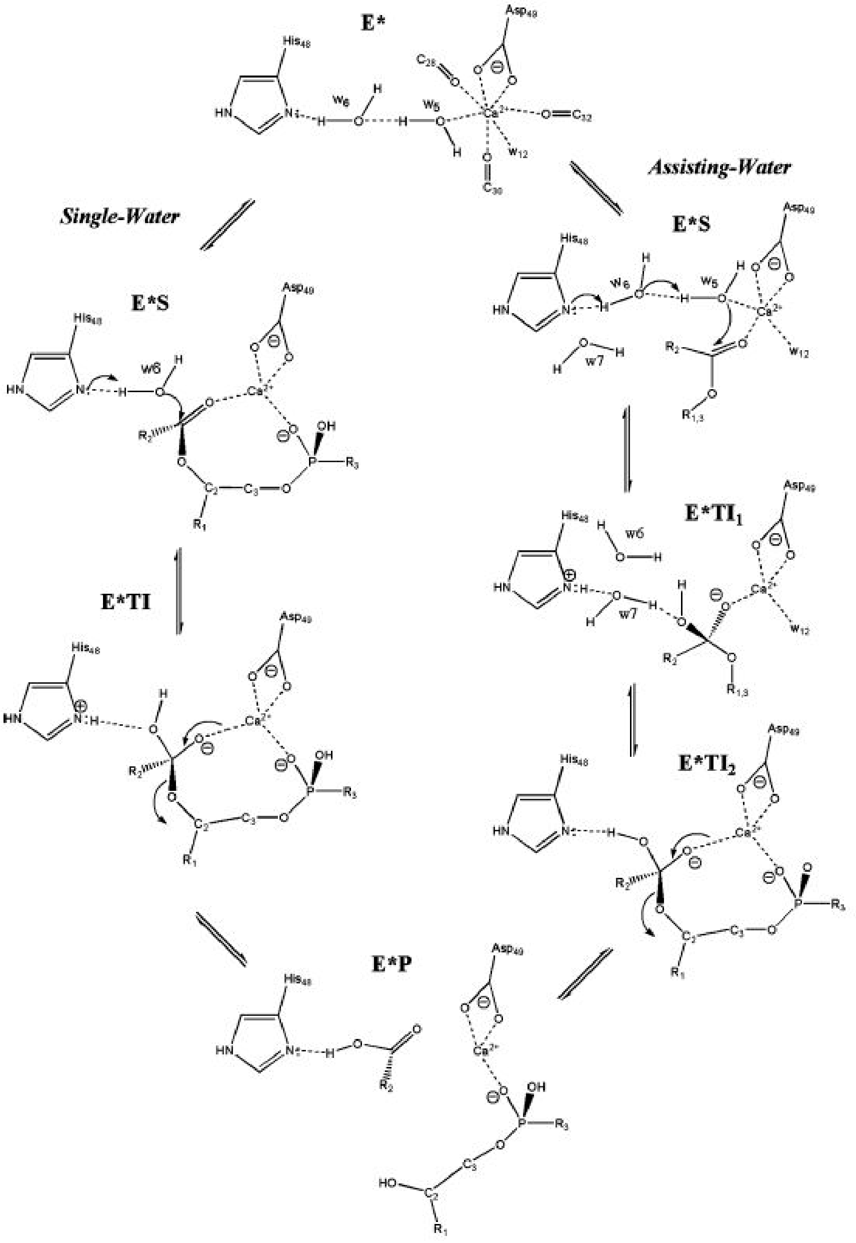

Figure S1: Two proposed catalytic mechanism for secreted PLA2s—the single-water and assisting-water mechanisms. "Original figure from Bahnson, 2005: Archives of Biochemistry and Biophysics, volume 433, pages 96–106, Elsevier, copyright 2005 [1]

Reference

1 Bahnson, B. J. Structure, function and interfacial allosterism in phospholipase A2: Insight from the anion-assisted dimer. *Arch. Biochem. Biophys.* 2005, *433*, 96–106.
